# Habitat suitability modelling of Koklass pheasant (*Pucrasia macrolopha*) in moist temperate forest

**DOI:** 10.1371/journal.pone.0296921

**Published:** 2024-02-15

**Authors:** Badrulislam, Kamal Ahmed Khan, Sangam Khalil, Majid Hussain, Zafeer Saqib, Javeria Altaf, Rana Hadi, Ume Habiba

**Affiliations:** 1 Department of Forestry and Wildlife Management, Faculty of Basic and Applied Science, The University of Haripur, Haripur, Pakistan; 2 College of Life Sciences, Shandong Normal University, Jinan, China; 3 Department of Forestry Range and Wildlife Management, The Islamia University Bahawalpur, Bahawalpur, Pakistan; 4 Department of Environmental Science, International Islamic University, Islamabad, Pakistan; 5 Department of Zoology, Government College University Faisalabad, Faisalabad, Pakistan; 6 Department of Zoology, Jinnah University for Women, Karachi, Pakistan; 7 Ministry of Climate Change, Islamabad Wildlife Management Board, Islamabad, Pakistan; Feroze Gandhi Degree College, INDIA

## Abstract

The decreasing status of on IUCN of Koklass pheasant (*Pucrasia macrolopha*) belongs to the family Phasianidae and the order Galliform needs the attention of researchers. The species with habitats as low as 6,000 feet and as high as 11,000 feet certainly cover a broad variety of habitats, such a wide altitude range embraces a diverse range of habitats. Insufficient research has been conducted on the suitability of moist temperate forests as a potential habitat for the Koklass pheasant. Therefore, this study was carried out to explore habitat suitability in 15 different sites which were located in the 4 districts of Hazara Division using GIS data science and environmental variables. A random sampling technique was used for laying out the transect. Overall, 45 line transects (Length 2–4 km, Width 10–30 m) were laid out in study sites. The size of sample plots for trees was 10x10m, for shrubs (4 x4m), and herbs and grasses 1x1m. The other habitat parameters like elevation, slope, cover, and frequency of plant at each point were also considered. We found the uneven distribution of Koklass pheasant in the Hazara Division. There were 59 occurrence points identified and highlighted the distribution of Koklass pheasant in the study area. Although all environmental variables were preferred by Koklass pheasant in its habitat statistical analysis proved that slope, level of disturbance, tree and shrub frequency of habitat contributed mostly to the presence of Koklass in each study site except the contribution of soil and herbs. The potential suitable habitat of Koklass pheasant was estimated to be 439.6 km^2^ areas starting from Abbottabad to Mansehra in the Hazara division. Awareness and enforcing legal protection are recommended for the conservation of Koklass Pheasant in Moist temperate forest.

## Introduction

Pheasants are habitat quality indicators as they are heavily dependent on understory and surface vegetation and sensitive to environmental quality fluctuations [1]. The single species Koklass (*Pucrasia macrolopha*) of the genus *Pucrasia*, belongs to the Phasianidae and order Galliform [2]. Koklass has wide global distribution and is native to Afghanistan, Nepal, India, and China. It occurs in the Himalayas of Afghanistan to Pakistan in the west, continuously through north India, Nepal, northeast Tibet, and China [3].

Koklass has spatial distribution and a viable population in Pakistan. It is distributed regionally in Kalam (Janshai, Batandar, Anakar) [4], Jammu, and Kashmir District (Hattian Bala) [5]. The habitat of Koklass pheasant is characterized by coniferous forest with a thick cover of understory shrub, ranging from 2200m to 3400m elevation [2]. Although there were possible habitat locations below 2500 m, there was the emphasis on the requirement for snow-free winter habitats. Due to a far lesser snowfall in the east of Nepal than in the western Himalayas, in winter the birds may not have to move towards low elevation [6].

The Koklass breeds in many areas from April to June [7]. This bird is thought to have a large global population and is presently considered a species of low conservation concern [8]. The nest of Koklass pheasant is usually composed of small sticks, feathers, twigs, and grasses [9]. Koklass pheasant describe as "extremely secretive and ultra-wary," and so its death rate as a direct result of human activity remains quite low [10]. The population trend of Koklass pheasant is decreasing [6]. The Koklass pheasant was found at a height of 9927 feet, indicating that the native habitat has been disturbed by human activity [4] causing the decline in species abundance [11]. The major threats to wildlife including birds are poaching, hunting, grazing, fuelwood collection, deforestation, disturbance, grass cutting, medicinal plant collection, which are the main factor leading to the degradation of biodiversity [12].

The moist temperate forest of Hazara division is vast and less explored in terms of habitat suitability of the Koklass pheasant (*Pucrasia macrolopha*). Few studies on different aspects of Koklass pheasant were found but the status of Moist temperate forest as a whole offer suitable habitat is missing.

This research was designed to study habitat suitability and preference modeling using Maxent Software (3.4.4) in the light of environmental variables to predict the future occurrence of Koklass pheasant in the study area. The results of this study will be helpful for the management and conservation of Koklass pheasant in the moist temperate forest of Hazara division.

## Materials and methods

### Ethics statement

The research conducted on habitat suitability modelling of Koklass pheasant (*Pucrasia macrolopha*) in moist temperate forest strictly adhered to ethical guidelines and was carried out with honesty, fidelity, and integrity. All measures were taken to ensure animal welfare, minimizing stress during capture and handling. The study complied with legal and ethical requirements, obtaining necessary permits before commencing fieldwork. Researchers minimized disturbance to the natural habitat, employed non-invasive methods, and ensured confidentiality of collected data while acknowledging contributions from stakeholders respectfully. The research team accepts full responsibility for their actions, committing only to endeavors they intend to follow through on, and did not engage in or participate in any type of malicious injury to other animals intentionally. This study was conducted with the primary aim of contributing ethically to Koklass pheasant conservation and enhancing understanding of their habitat.

### Study area

The Hazara division i.e., study area is located in Pakistan’s Khyber Pakhtunkhawa province, in the northeastern part of the country. It consists of seven districts i.e., Haripur, Abbottabad, Battagram, Manshera, Upper Kohistan, Lower Kohistan, and Torghar which are east of the Indus River. Hazara division is 18012 km^2^ in size and the total study area of moist temperate forest is 1470 km^2^. Regarding climatic factors; the annual mean temperature ranges from 13.96°C to 15.73°C is recorded moving towards the north to the southern part.

The mean daily temperature reaches 12.7°C in June (before the monsoon) and the temperature decreases to 3°C in January. Similarly, annual precipitation (mm) ranges from 1,145 to 1,376 mm moving from the north to the southern part. Overall, maximum rainfall of 355 mm is observed in August (monsoon) and minimum (17.9 mm) in November after monsoon months. The moist temperate forests extended over an elevation range from 1,830 to 2,972 m but most of the track falls in the 2,150–2,910 m range [13]. Similarly, slope range from medium to steep slope (up to 72 degrees) throughout moist temperate forest range while Ecotone area has gentle slope comparatively.

The major flora was Conifers, in particular Gowargi (*Pinus wallichiana*), Achar *(Abies pindrow)*, Dear *(Cedrus seodara)*, Kachal *(Picea smithiana)*, Chir Pine (*Pinus soxburghii*), Bankhor *(Aesculus indica)*, *Yew (Taxus baccata)*, Oak *(Quercus incana)*, Walnut (*Juglans regia*), Moru Oak (*Quercus floribunda*), Ring-Cupped Oak (*Q*. *glauca*) and Deciduous broad-leaved trees Himalayan Maple (*Acer caesium*), Kandara (*Cornus microphylla*), Walnut (*Juglans regia*), Himalayan Poplar (*Populus ciliate*), Himalayan Bird Cherry (*Prunus cornuta*), Willow (*Salix tetrasperma*) and Himalayan Elm (*Ulmus wallichiana*) [2, 14, 15]. Some major faunal species was Murree Vole *(Hyperacrius wynnei*), Porcupine (*Hystrix indica*), Rhesus Monkey (*Macaca mullata*), Common Leopard (*Panthera pardus*), Leopard Cat (*Prionailurus bengalensis)*, Koklass Pheasant (*Pucrasia macrolopha*), Kalij Pheasant (*Lophura leucomelana*), Jungle Crow (*Corvus macrorhynchos*), and Tree Pie (*Anthus trivialis)* [14].

### Research design

A reconnaissance survey of the research region, namely the Hazara division’s Moist Temperate Forest, was undertaken, and prospective Koklass Pheasant habitat sites were found. The Koklass pheasant was distributed on four research sites, which were further split into 15 study sites (Transects), which were used as wide sample units in the current study. The presence of the Koklass pheasant was established either directly or indirectly (feather, calls). The occurrence of the Koklass pheasant was recorded to create a distribution map. ArcGIS software version 10.3 was used to create the map [3, 12, 13].

During 2020–2021, each selected research site was sampled for floral diversity and community structure by measuring diverse tree, shrub, and herb species to evaluate the habitat use of Koklass Pheasant. The distribution of Koklass Pheasant was determined using the quadrat technique along a transect line.

Each study site utilized a varied length of the transect, with each sample point randomly spaced apart. A total of 150 quadrats were collected by placing ten (10) quadrats perpendicular to a straight line at each study site. In each quadrate, the density, frequency, and percent cover of each plant species were recorded. Sample plots for trees (10m x 10m), shrubs (4m x 4m), and herbs (1m x 1m) were chosen [8].

At each sample location, the terrain, slope, water points, and amount of disturbance were all recorded as physical characteristics of the ecosystem. Elevation and aspects were also recorded using the Global Positioning System (GPS) and Marching Compass. The slope was determined using a clinometer. Plant specimens were collected, pressed, dried, and mounted on herbarium sheets. The accurate identification of the gathered plants was followed by a study of the flora of Pakistan [16].

The plants were matched to previously recognized plants in the University of Swat’s herbarium. Locals were invited to come up with vernacular names for species. Density, relative frequency, and relative coverage were calculated using the following formula [17].


$$Density\;(D)=\frac{Total\;No.\;of\;individuals\;of\;a\;species}{Total\;area\;sampled}$$

$$Relative\;Frequency\;(RF)=\frac{No.\;of\;quadrats\;in\;which\;species\;occur\;x\;100}{Total\;No.\;of\;quadrates}$$

$$Relative\;cover\;(RC)=\frac{Cover\;of\;individuals\;of\;a\;species\;x\;100}{Total\;cover\;of\;all\;individuals\;of\;all\;species}$$

To create a habitat suitability model for Koklass in the moist temperate forest of Hazara division. Version 3.4.4 of the Maxent software was used [18]. The maximum entropy model (Maxent) is one of the most widely used java-based maximum-entropy approaches [18, 19].

The ANOVA test was applied to check the difference in the occurrence of direct and indirect signs at different locations (study sites). One-way ANOVA was applied to check the presence of environmental variables.

## Results

The study area was searched for 6 months and 15 potential habitat areas named study sites were selected based upon information gathered from employees, and local people about its occurrence.

Fifty-nine (59) direct and indirect signs were collected from a study comprising four districts of the moist temperate forest of Hazara division. Upon the occurrence of signs, data revealed that the Koklass pheasant was not evenly distributed in the study area. Koklass pheasant was recorded directly in 4 out of 15 study sites (Table 1).

**Table 1:** Characteristic of study site in the moist temperate area of Hazara division.

S. No	District	Name of study Area	Altitude	Longitude	Elevation (m)	Slope (%)	Area of Transect (km^2^)
1	Abbottabad	Khanaspur	34.02591	73.42974	2000–2500	40	4
2		Mashkpori	34.05807	73.42569	2400–2800	36.7	6.4
3		Donga gali	34.04173	73.39306	2200–2800	47	3.6
4		Thandiyani1	34.23771	73.35539	2300–2700	33.6	5
5		Thandiyani2	34.24098	73.37379	2300–2900	49	2.2
6	Manshera	Kannia	34.73212	73.49206	2100–2800	34.4	7
7		Kannia 2	34.7263	73.48538	2300–3000	34.5	2
8		Kamal band	34.70606	73.54047	2200–2800	31.7	5.6
9		Siripae	34.91451	73.48682	2400–2900	26.7	5.2
10	Battagram	Ganthar	34.89645	73.19006	2300–2800	42.4	5
11		Shamsher	34.90232	73.19462	2300–2800	41.5	5
12		Battangi Band	34.91914	73.08314	2500–3000	37.9	6
13	Torghar	Jatka	34.57438	72.90055	2300–2800	31.5	4.32
14		Kalapahar	34.51775	72.90991	2200–2900	29.9	5.6
15		Machai sar	34.58808	72.90909	2400–3000	36.7	6

**Fig 1:**
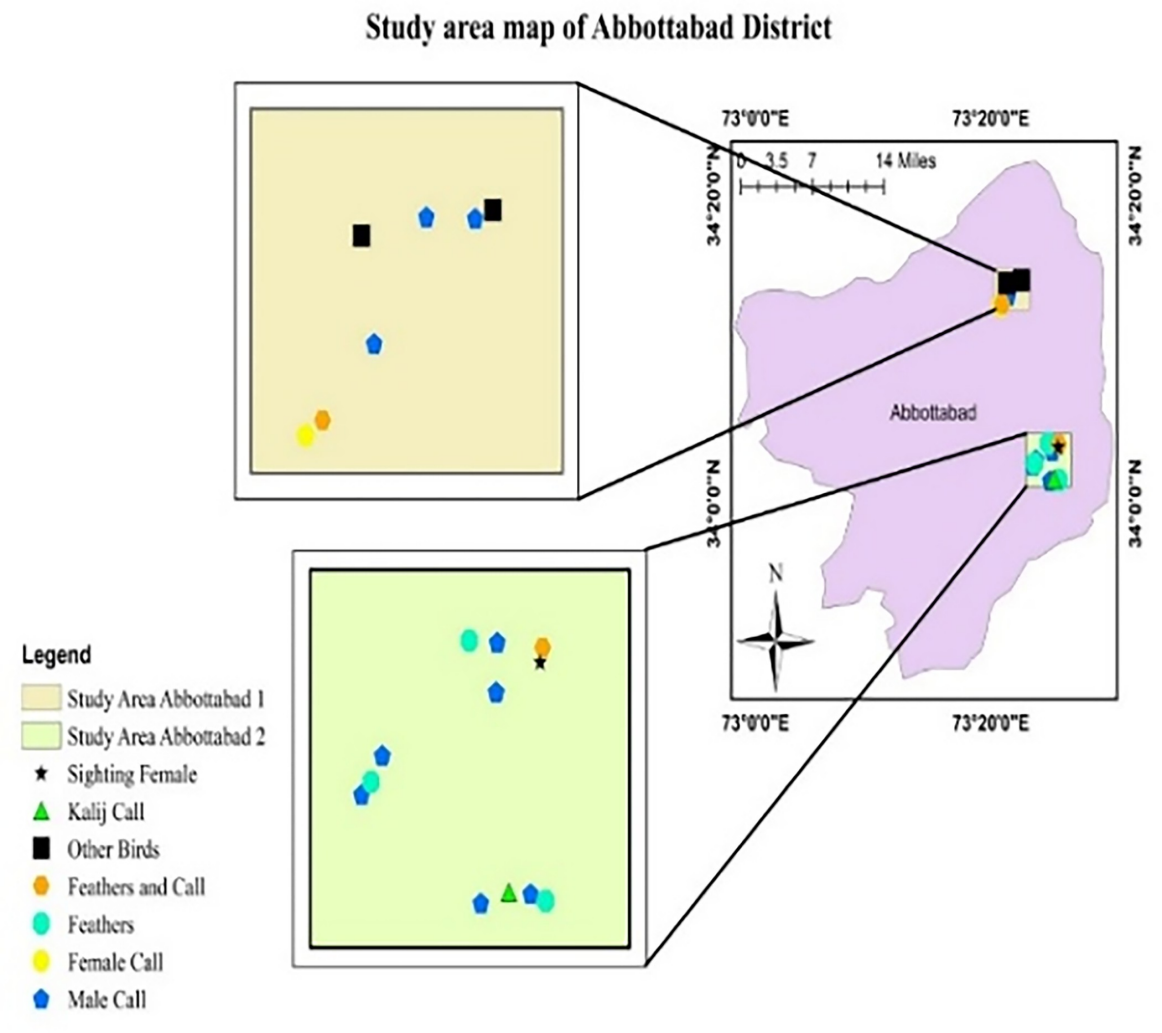
Occurrence signs of Koklass in District Abbottabad.

**Fig 2:**
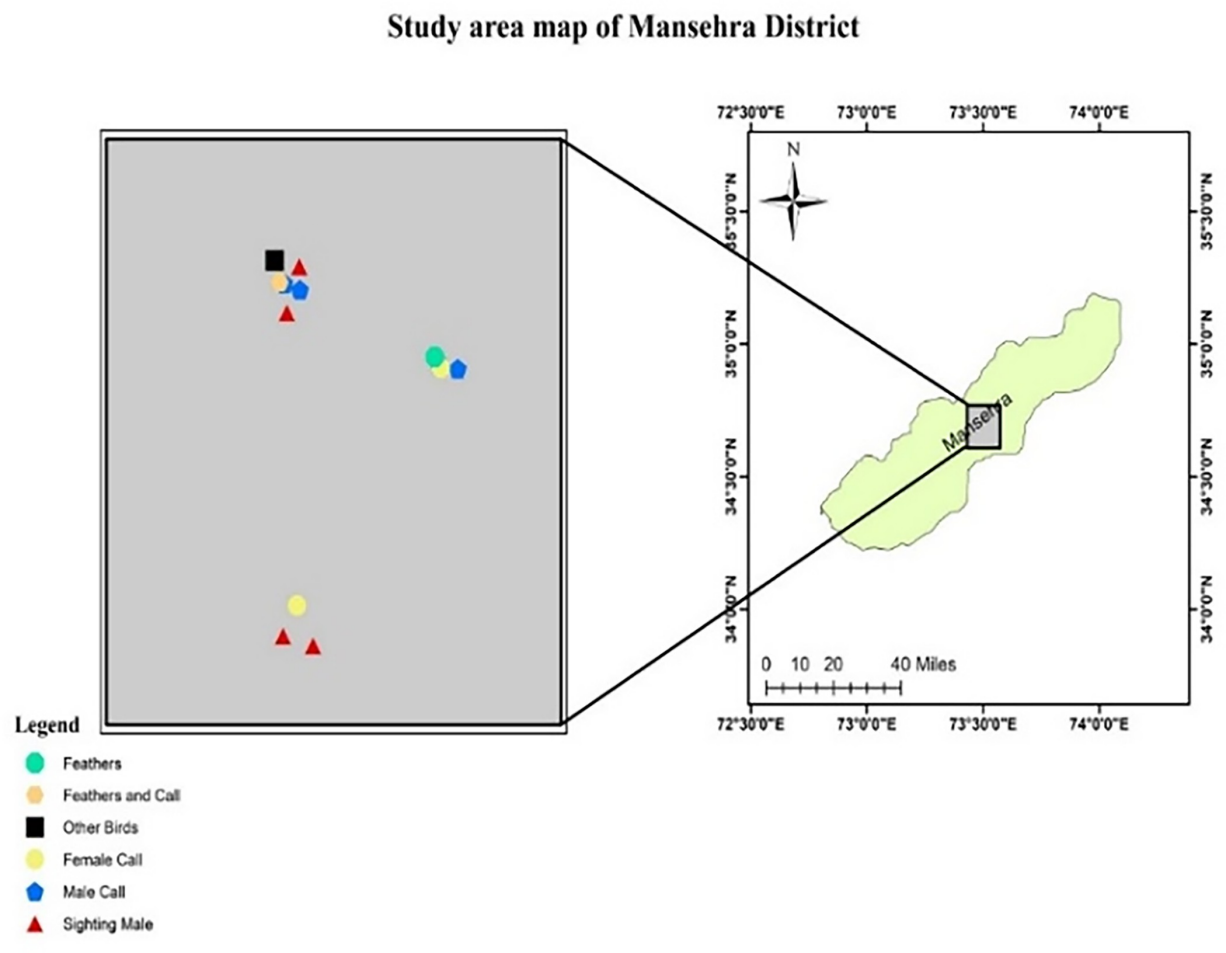
Occurrence signs of Koklass in District Manshera.

**Fig 3:**
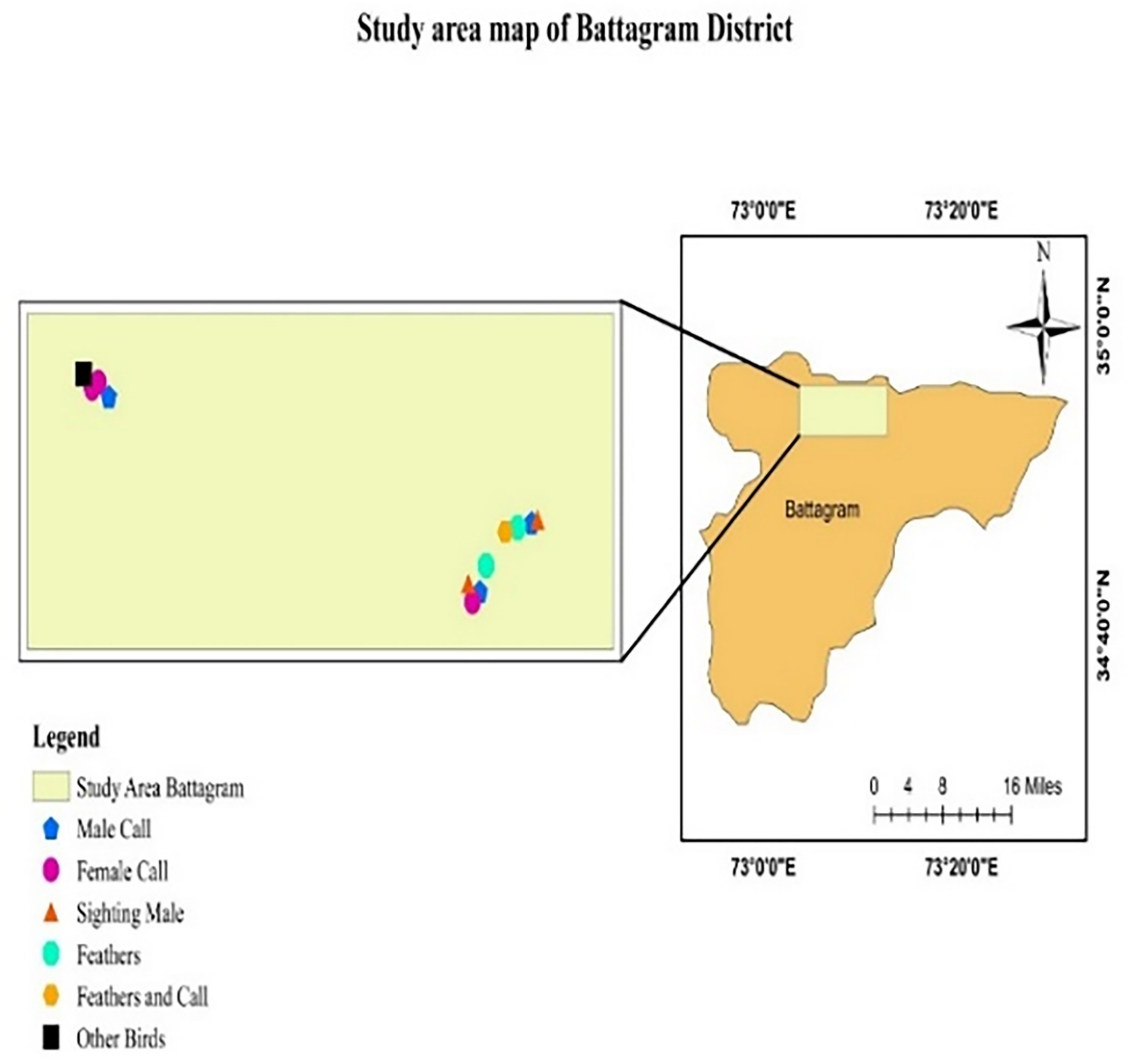
Occurrence signs of Koklass in District Battagram.

**Fig 4:**
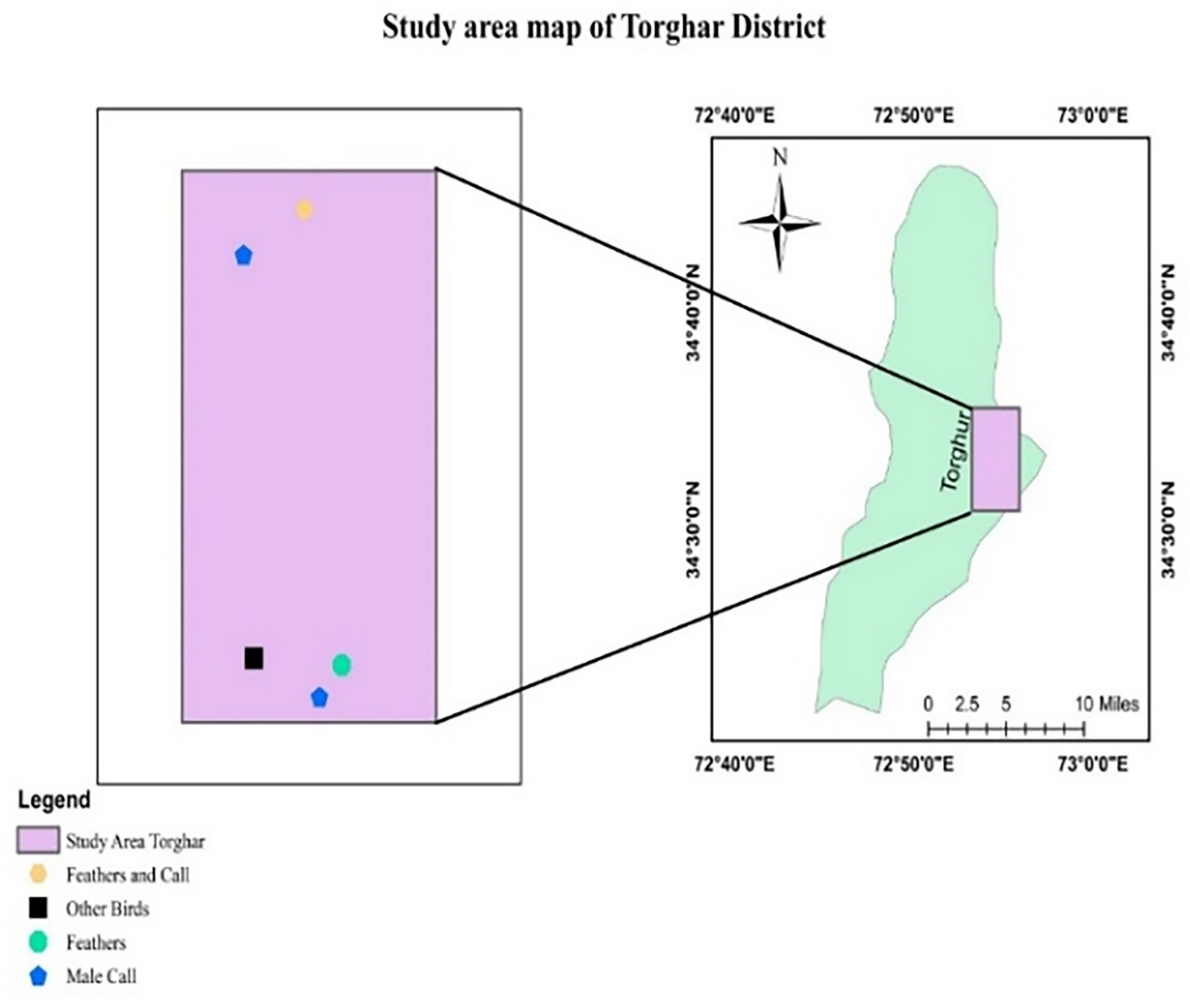
Occurrence signs of Koklass in District Torghar.

While indirect evidence (feather and call) was recorded in all study sites (Figs 1–4).

The ANOVA test showed that the value of P = 0.19 > α = 0.05 so there is no significant difference in presence of signs in any districts. All study sites were equally occupied by the Koklass pheasant (Table 2).

**Table 2:** Occurrence pattern of signs at different study sites.

Area	Sum of squares	df	Mean square	F	P-value
Abbottabad	9.893	5	1.979	0.32	0.898
Manshera	18.613	4	4.653	0.796	0.534
Battagram	1.201	3	0.4	0.066	0.978
Torghar	27.47	3	9.157	1.656	0.19

ANOVA test shows (Fig 3) that P-value is greater than α 0.05. So, there is no difference in presence of signs in all study sites.

**Fig 5:**
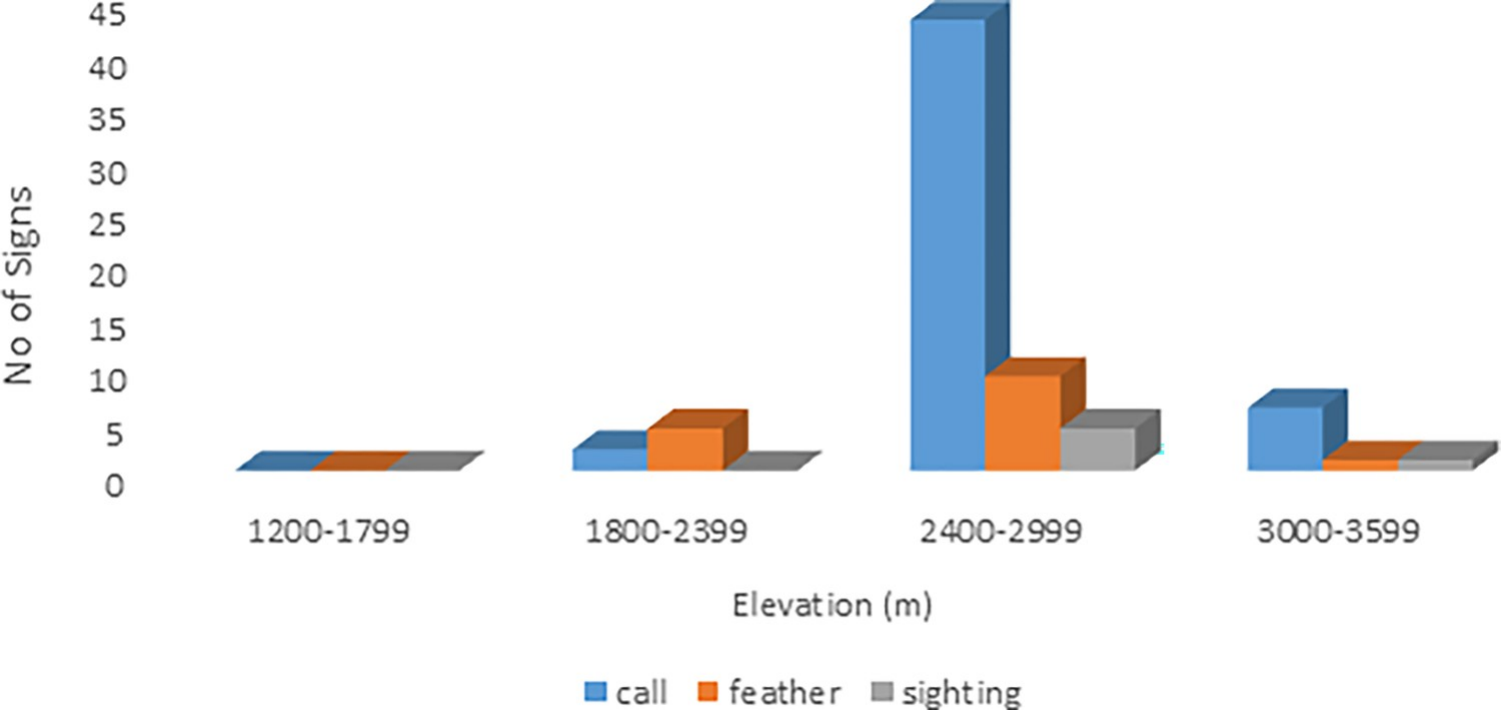
Signs of occurrence at different elevations.

Most of the direct observations were recorded in dense vegetation cover of trees during the present study. The species are mainly found on the elevation 2400 m to 3000 m in the study area (Fig 5).

The ANOVA test showed that slope, level of disturbance, tree frequency, and shrub frequency of habitat parameters contributed in presence of Koklass in each study site. Although the contribution of soil and herbs is less (Table 3).

**Table 3:** ANOVA test of the presence of signs with the environmental variable in the habitat of Koklass pheasant.

Variables	Sum of Square	df	Mean Square	F	P-value
Slope	2092.537	14	149.467	2.152	0.03
Soil	6.787	14	0.485	1.322	0.244
Level of disturbance	12.417	14	0.887	2.285	0.024
Tree frequency	149.837	14	10.703	2.17	0.032
Shrub frequency	79.513	14	5.68	2.593	0.011
Herb frequency	60.667	14	4.333	1.002	0.472

At elevations of 2,400–4,500 m, the species is found in temperate conifer and oak woods interspersed with open grassy slopes, cliffs, and alpine meadows in the Himalayan areas of Afghanistan, Pakistan, India, China (Tibet region), Nepal, and Bhutan [20]. The Koklass pheasants may be found across their range in the Himalayas between 1300 and 3000 meters [21].

**Fig 6:**
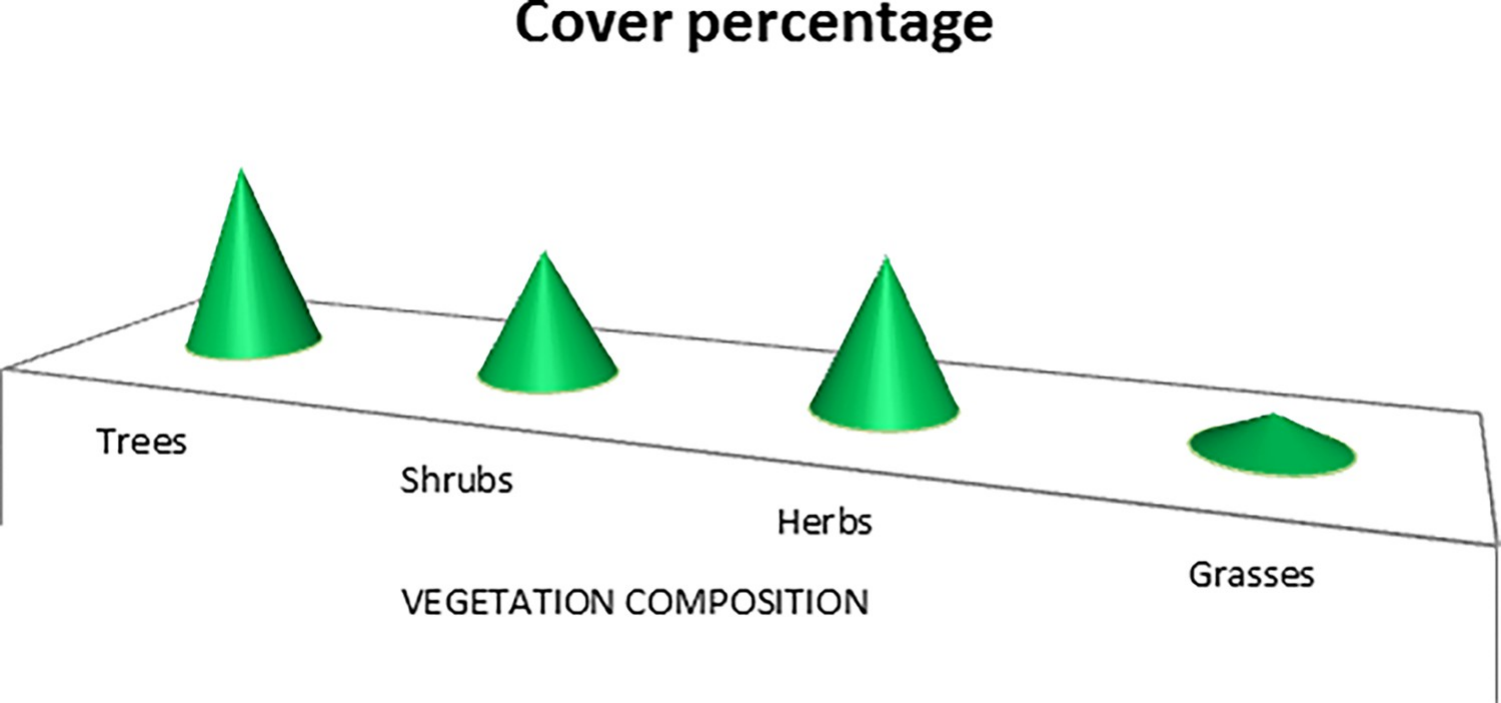
Percentage of vegetation cover in study area of Koklass pheasant.

The Koklass pheasant’s habitat comprised of 69 plant species (trees = 10, shrubs = 35, herbs = 18, and grass = 6), (Table 4). According to data on the distribution of vegetative cover of trees provided by various species in potential habitat areas (Fig 6).

**Table 4:** Analysis of plant species found in Koklass pheasant habitat.

S. No.	Plant Species	Density	Relative Frequency	Relative Cover
**Trees**				
1	*Abies pindrow*	3.22	96	30.37
2	*Picea glauca*	2.81	94	30.86
3	*Pinus wallichiana*	2.4	68	29.39
4	*Cedrus deodara*	0.2	19.3	2.93
5	*Taxus wallichiana*	0.37	30	1.7
6	*Populous ciliate*	0.07	6	0.7
7	*Juglans regia*	0.2	16	1.46
8	*Quercus dilatata*	0.06	6.6	0.5
9	*Betula utilius*	0.09	8.6	1.04
10	*Aesculus indica*	0.1	6	0.7
**Shrubs**				
1	*Parrotiopsis jacquemontiana*	0.19	7.3	4.86
2	*Viburnum cotinifolium*	1.77	26	29.47
3	*Asteraceae spp*.	0.34	10.6	2.57
4	*Geranium wallichiana*	0.19	8.6	1.57
5	*Skimmia laureola*	0.2	12.6	1.14
6	*Rubus fruticosus*	0.11	6	1.2
7	*Sarcococca saligna*	0.08	4	2.43
8	*Dryopteris ramosa*	0.19	6	2
9	*Lamium album*	0.2	9.3	1.85
10	*Isodon rugosus*	0.14	3.3	3
11	*Pteris cretica*	0.12	4.6	2.71
12	*Punica graatum*	0.12	7.3	1.14
13	*Rubus spectabilis*	0.16	7.3	1.28
14	*Plectranthus rugosus*	0.11	6	2.43
15	*Sorbarira tomentosa*	0.14	4.6	2.71
16	*Ribes alpestre*	0.19	6	4
17	*Indigofera heterantha*	0.2	6	3.43
18	*Calamintha umbrosa*	0.14	10.6	3
19	*Tagotes spp*.	0.06	4	0.78
20	*Clematis vitalba*	0.04	3.3	0.57
21	*Rosa webbiana*	0.1	2.6	1.28
22	*Berberis lycium*	0.32	5.3	6.86
23	*Andranchne cordifolia*	0.28	12	4.86
24	*Rosa macrophylla*	0.06	2.6	0.85
25	*Maytenus ruffa*	0.04	3.3	0.42
26	*Lonecers spp*.	0.11	2.6	1.28
27	*Jasminum humile*	0.06	2	1
28	*Jasminum grandiflorum*	0.06	4	0.64
29	*Barleria*	0.09	4	0.85
30	*Rubus ellipticus*	0.05	4	1.71
31	*Cotoneaster*	0.14	9.3	1.57
32	*Artemisa valgaris*	0.12	4.6	2.57
33	*Saussurea lappa*	0.07	4	1.14
34	*Tracheophyta*	0.08	4	1.43
35	*Clematis vitalba*	0.09	2.6	1.26
**Herbs**				
1	*Plantago osiatica*	0.33	12	13.6
2	*Gloriosa superba*	0.12	6.6	4.04
3	*Asplenium filix*	0.18	9.3	13.9
4	*Ranunculus muricatus*	0.11	9.3	2.52
5	*Sinapis arvensis*	0.11	4	2.52
6	*Micromeria biflora benth*	0.09	4.6	3.03
7	*Bistorta amplexicaulis*	0.2	5.3	9.1
8	*Ephorbia helioscopia*	0.16	11.3	2.9
9	*Adiantum capillus-veneris*	0.13	1.3	3.1
10	*Hedera nepalensis*	0.09	7.3	3.4
11	*Malva neglecta*	0.06	1.3	1.39
12	*Artemisia valgaris*	0.29	9.3	14.2
13	*Polypodisopsida*	0.16	12	4.93
14	*Solidago virgaurea*	0.16	7.3	3.53
15	*Rumex dentatus*	0.19	6	6.57
16	*Artemisia absinthium*	0.14	11.3	3.28
17	*Oxalis corniculatus*	0.11	9.3	3.53
18	*Cynodon dactylon*	0.17	8	4.1
**Grasses**				
1	*Tbemeda triandra*	2.4	26.6	29.7
2	*Cyperus rotundus*	2.5	23.3	30.2
3	*Cbrysopogon gryllus*	1.6	14	9.09
4	*Dactylis glomerata*	2.4	17.3	12.3
5	*Koeleria macrantha*	1.9	17.3	11.2
6	*Andropogon munroi*	0.7	10.6	7.4

### Trees

**Fig 7:**
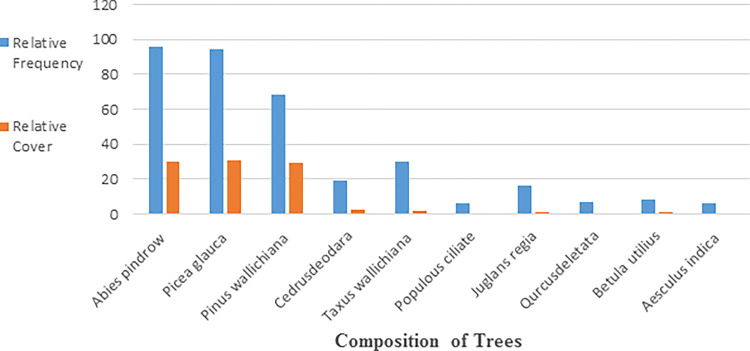
Relative frequency and cover of different trees species in study area.

The density of tree species per quadrat was compared (Table 4). The data showed that among all tree species *Abies pindrow* was the most dominant species followed by *Picea glauca* and *Pinus wallichiana*. Occasional and rare species comprised *Cedrus deodara*, *Taxus walliciana*, *Populous ciliate*, *Juglans regia*, *Qurcus deletata*, *Betula utilius*, *and Aesculus Indica* (Table 4). *Abies pindrow and Picea glauca* surpassed all other species having a frequency of 96 and 94, respectively. The least frequent was *Aesulu sindica* 6 (Fig 7).

*Picea glauca* and *Abies pindrow* covered the major area of vegetation cover among tree species comprised of (30.86, 30.37, and 29.39). The average cover of other species ranges from 0.5 to 2.93% (Table 4).

### Shrubs

**Fig 8:**
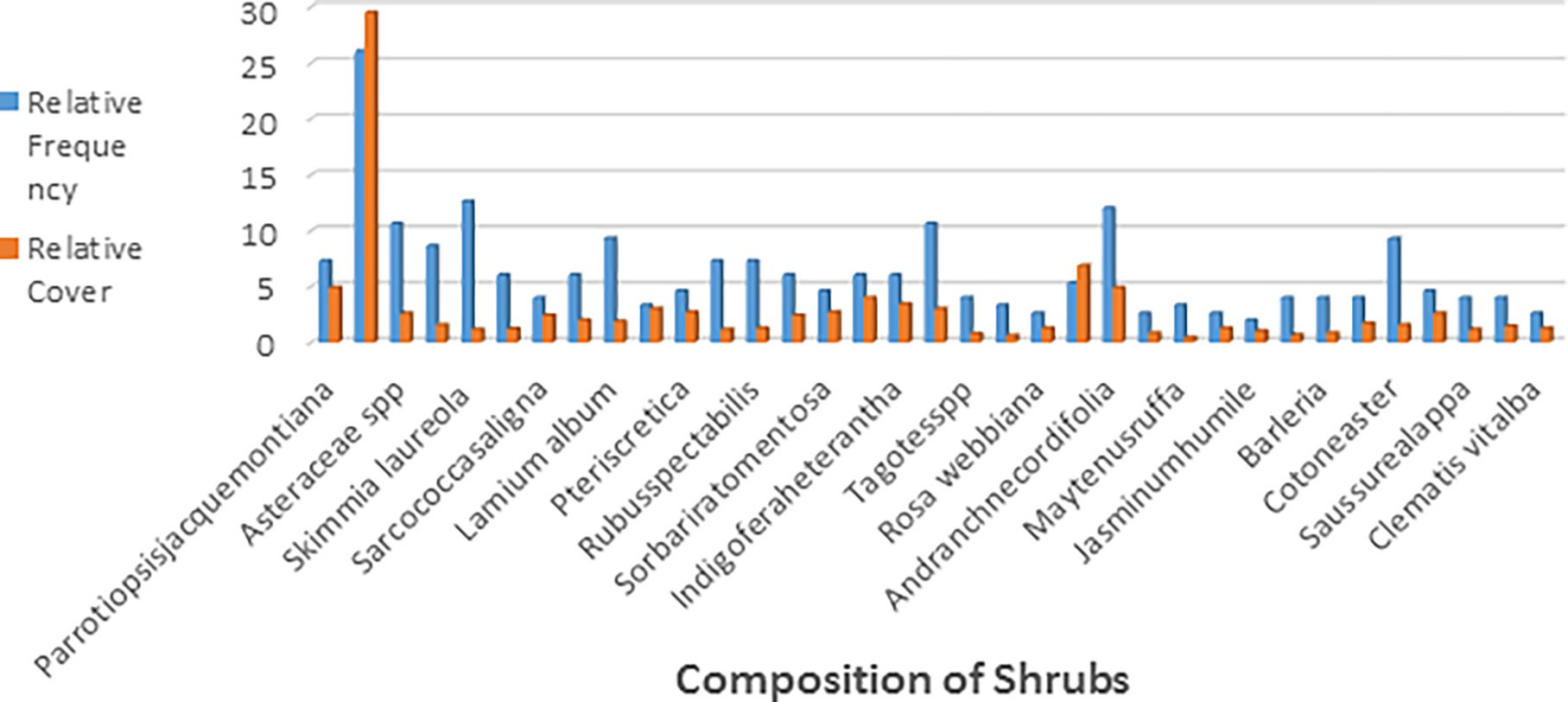
Relative frequency and cover of different shrubs species in study area.

Among shrub species, *Vibernum cotinifolium*, *Asteraceae Spp*., *Bereris lyceum*, and *Andranchne* were the most densely growing species. Other rare species were included in (Table 4). *Vibernunm cotinifolium* was the most frequent species having a frequency is 26, respectively. Less frequent species recorded in the habitat of Koklass pheasant included *Punica graatum* (7.3), *Rubus spectabilis* (7.3), *Dryopteris ramose* (6), and *Rubus fruticosus* (6) and other rare species include in (Fig 8).

*Vibernunm cotinifolium* covered a major area of vegetation cover among shrub species comprising 29.47%. Some species have minor vegetation cover ranging from 1 to 4% (Table 4).

### Herbs

**Fig 9:**
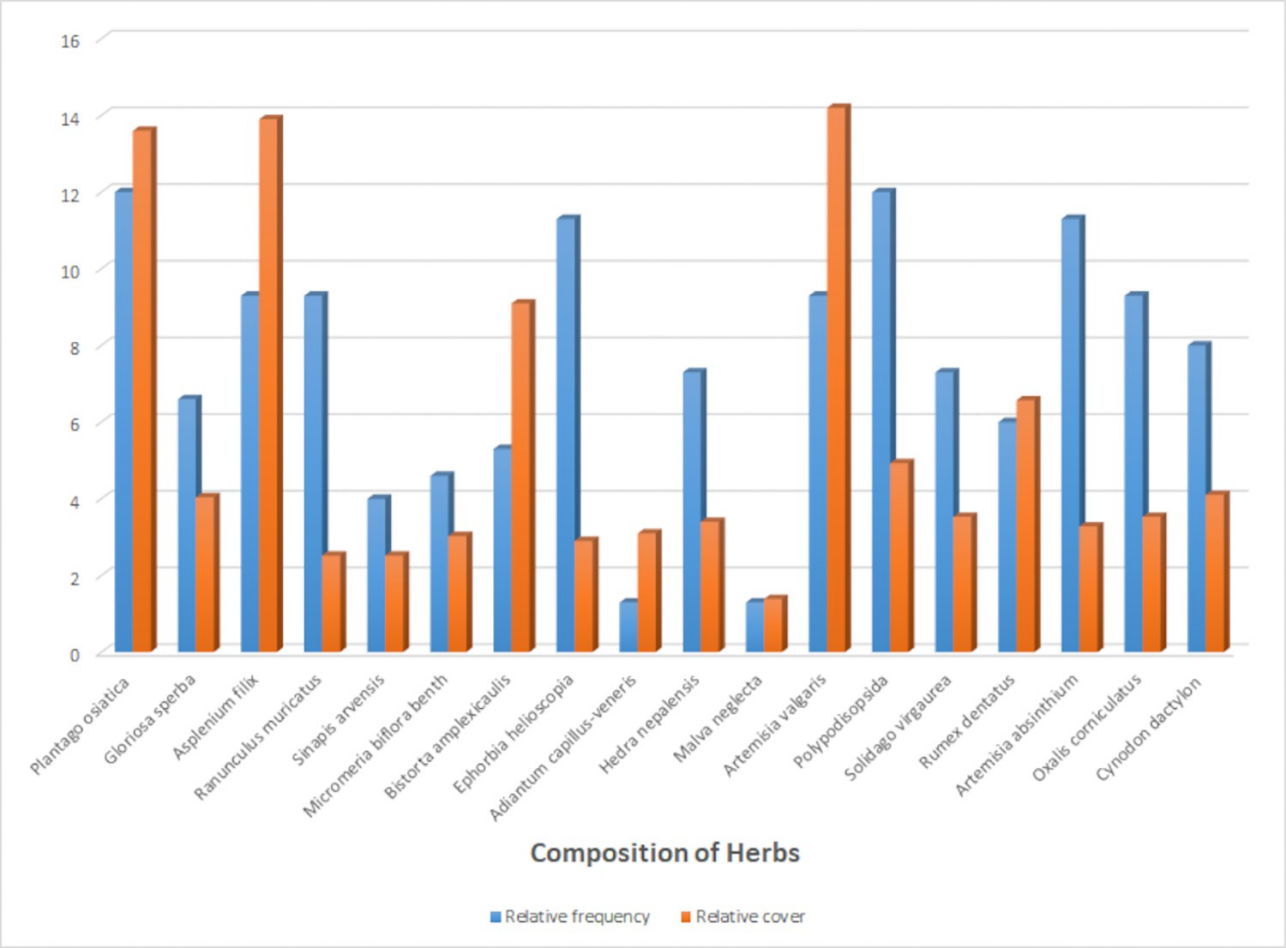
Relative frequency of different species of herbs in study area.

*Plantago osiatica* and *Artemisia valgaris* were the most commonly found species. Rarely occurring herbs were mentioned in (Table 4). *Polypodiopsida* (12) *Plantago osiatica* and (12) were found most frequent among all herbaceous. Some other rare species were included in (Fig 9).

*Artemisia vulgaris* covered a major area of vegetation cover among herb species (14.2%). Other rare species had low vegetation cover less than 1.3% (Table 4).

### Grasses

**Fig 10:**
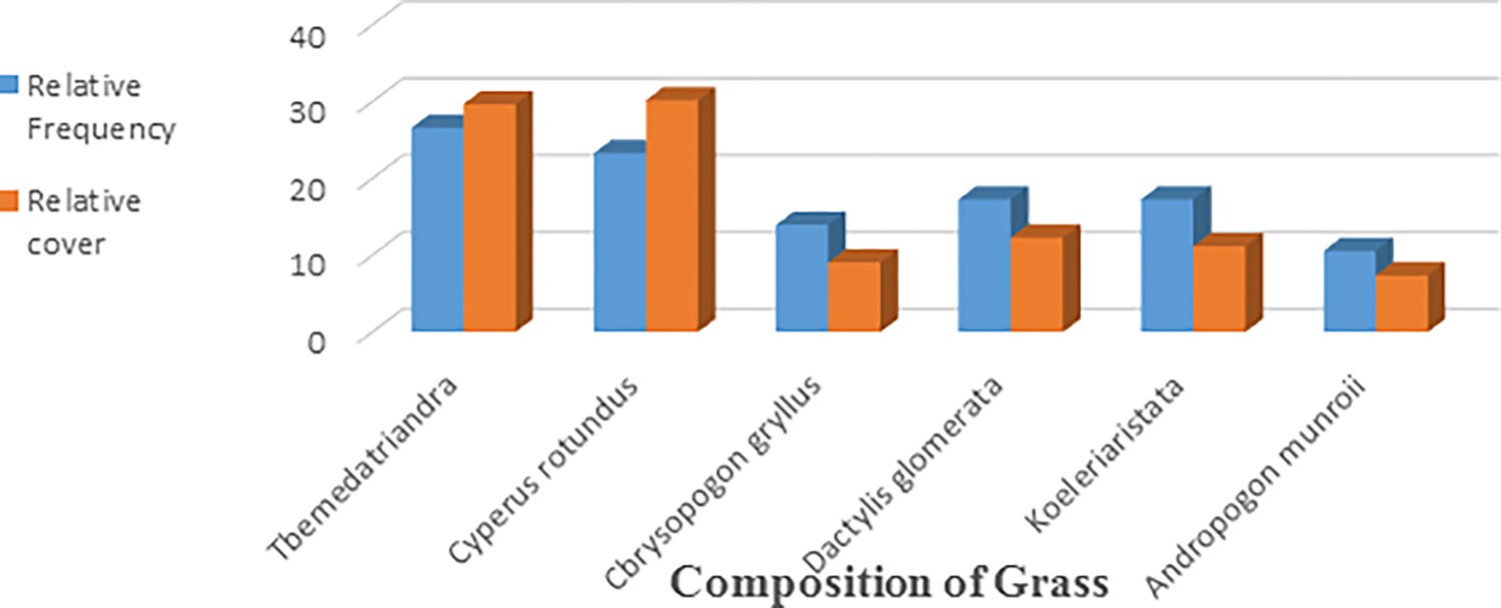
Relative frequency of different species of grass in study area.

*Cyperus rotundus* was the most commonly found grass species. Rarely occurring grass was *Andropogon munroi* (Table 4). *Tbemeda trindra*, *Cyperus rotundus*, and *Koeleria cristata* were found most frequent among all grass species, closely followed by *Cbrysopogon gryllus* and *Andropogon munroi*. *Cyperus rotundus* covered a major area of vegetation cover among grass species. Followed by *Tbemeda trindra*, *Dactylis glomerata*, *Koeleria cristata*, etc. (Fig 10).

### Maxent modelling

To create a habitat suitability model for Koklass in the moist temperate forest of Hazara division. Version 3.4.4 of the Maxent software was used [18].

**Fig 11:**
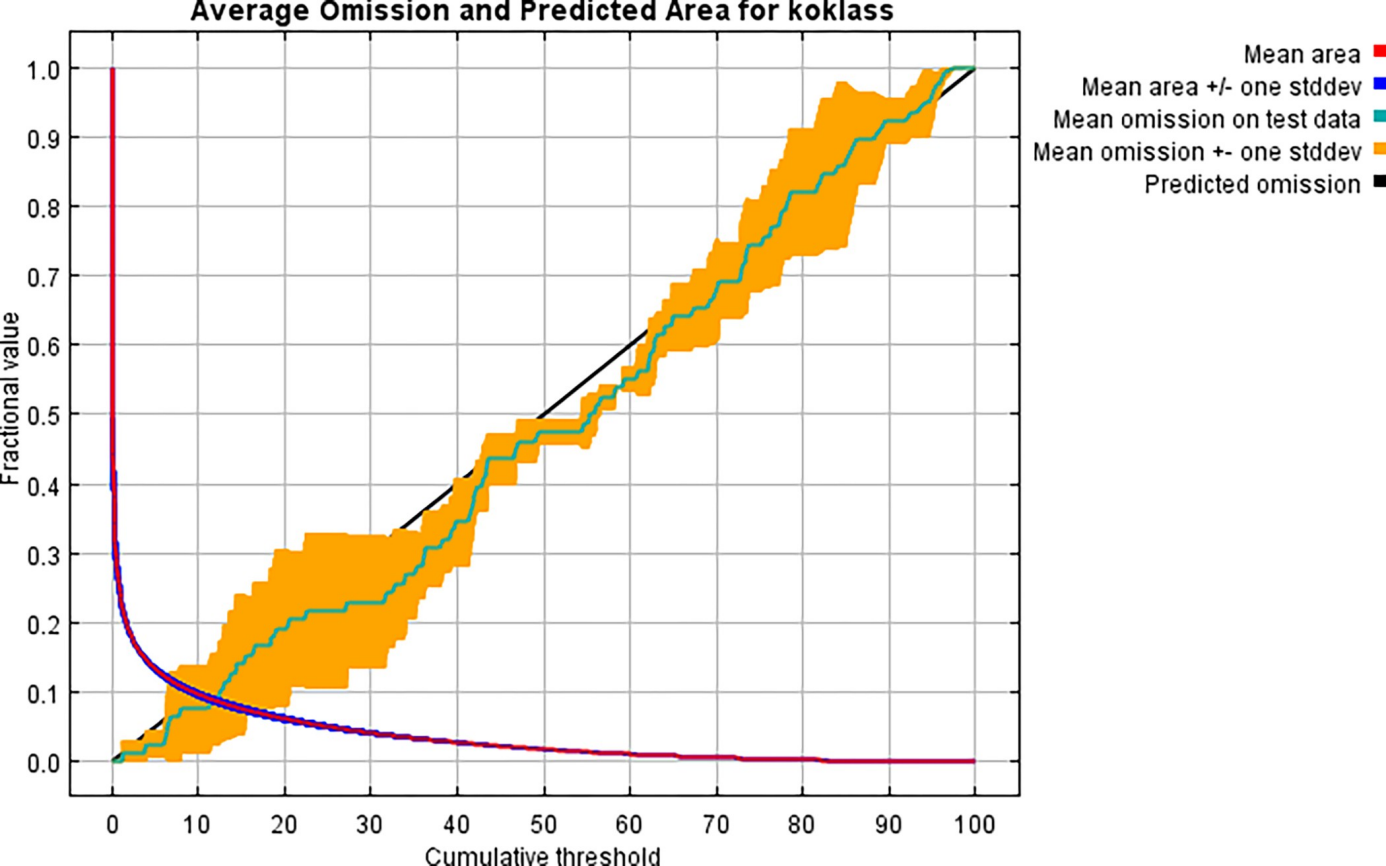
The area under the curve (AUC) of fractional value vs. cumulative threshold of Koklass habitat for Maxent model.

The test omission rate and projected area as a function of the cumulative threshold averaged across the duplicate runs, are shown in the graph below. Because the cumulative threshold is defined, the omission rate should be close to the predicted omission (Fig 11).

**Fig 12:**
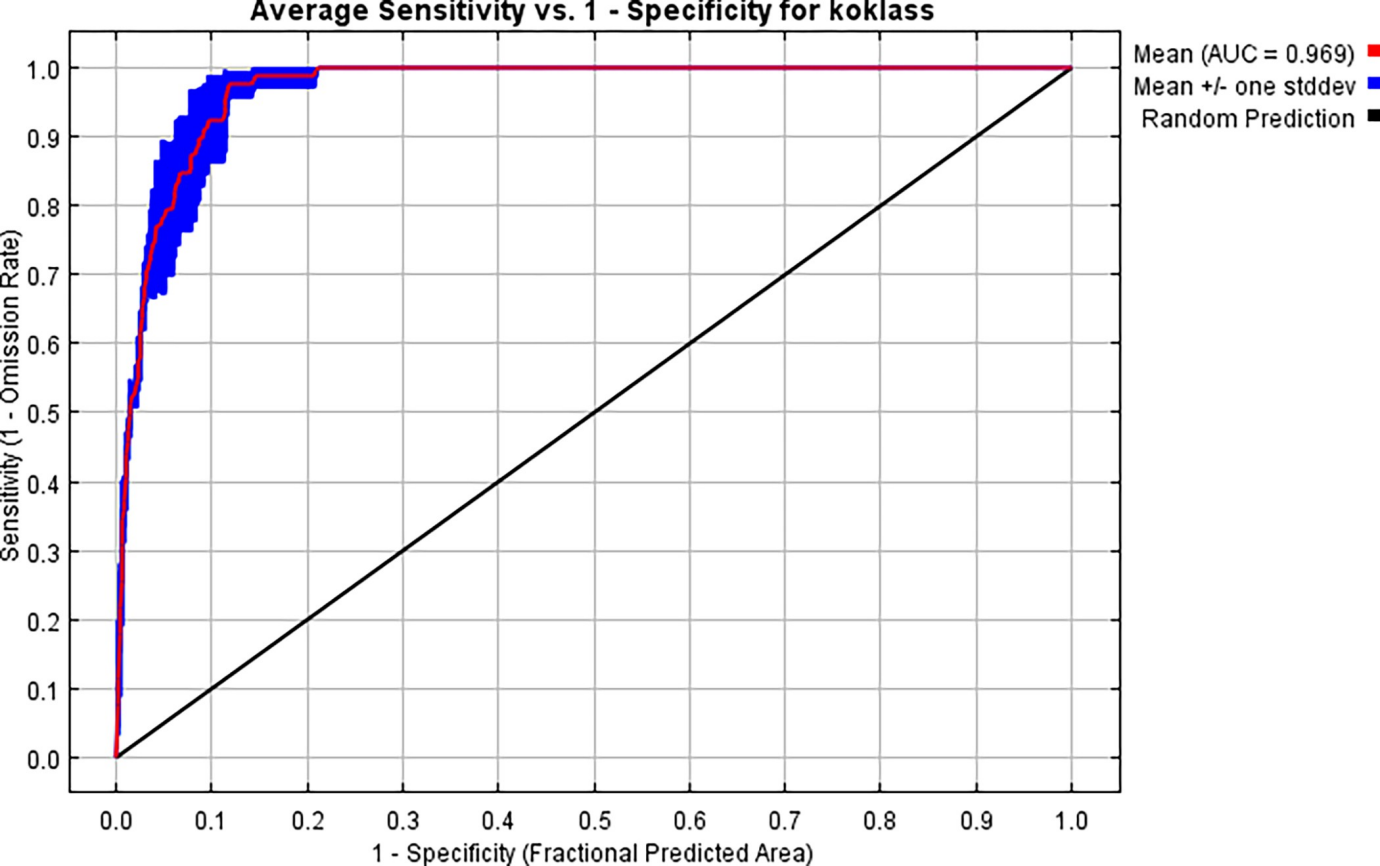
Receiver operator characteristic (ROC) curve of sensitivity vs. specificity of Koklass habitat for Maxent models.

The receiver operating characteristic (ROC) curve for the same data is shown in (Fig 12), which has been averaged over the duplicate runs once more. It’s worth noting that the specificity is determined by the projected area rather than the actual commission. The standard deviation is 0.007, and the average test AUC for the replicate runs is 0.969.

**Fig 13:**
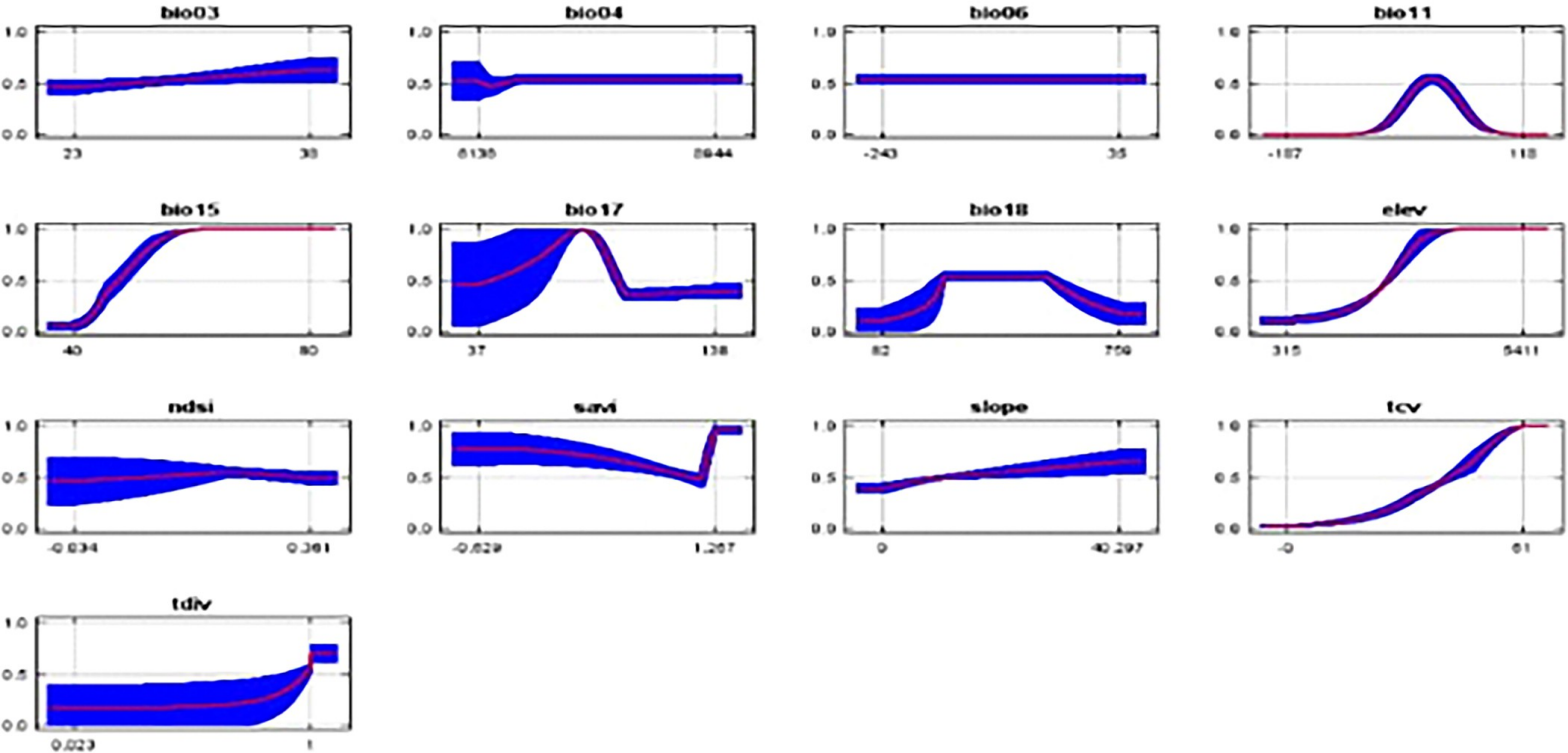
Contribution of environmental variables in modelling.

These graphs demonstrate how each environmental variable influences the Maxent forecast (Fig 13).

The graphs depict how the expected probability of presence changes as each environmental variable is changed while all other environmental variables remain constant. If you have heavily correlated variables, the curves might be difficult to interpret since the model may be dependent on the correlations in ways that aren’t visible in the curves. To put it another way, the curves depict the marginal effect of altering only one variable, but the model may benefit from several variables changing at the same time. The mean response of the three replicates Maxent runs (red) and the mean +/- one standard deviation are shown in the graphs (blue, two shades for categorical variables) (Table 5).

**Table 5:** Estimation of contribution of the environmental variables of maxent models.

Variable	Percent contribution	Permutation importance
tcv	70.4	11.4
bio18	7.6	9.9
bio06	5.5	0
bio17	5.1	6.3
bio03	3.9	0.1
tdiv	2.7	4.2
savi	1.5	0.3
bio11	1	43.9
bio15	0.9	10.7
elev	0.7	12.6
slope	0.5	0.2
ndsi	0.2	0.1
bio04	0	0

**Fig 14:**
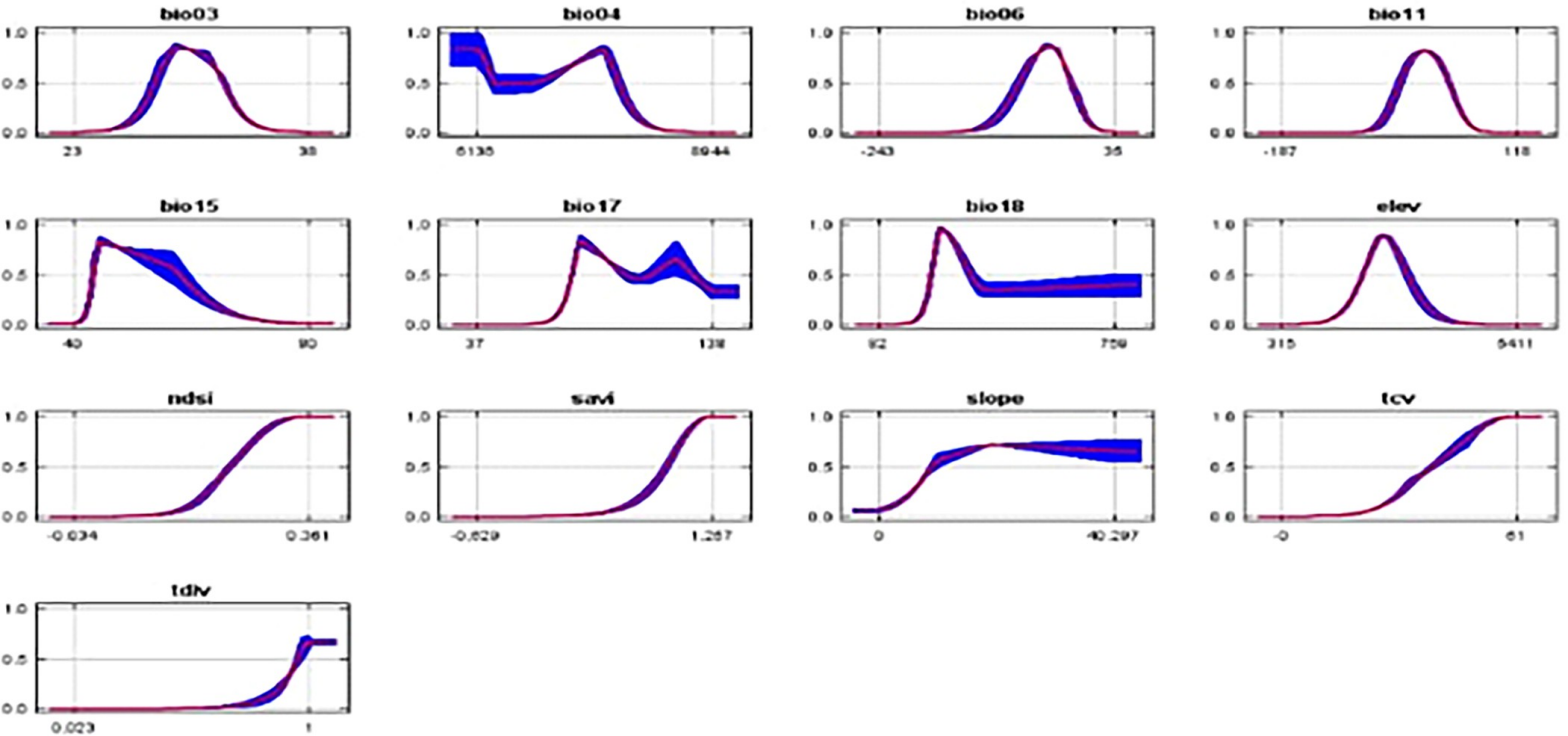
Contribution of environmental variables in modelling.

Unlike the marginal response curves before, each of the curves below represents a separate model, particularly a Maxent model built using only the associated variable. These graphs show how projected appropriateness is dependent on the selected variable as well as on dependencies caused by correlations between the selected variable and other factors. If there are significant correlations between variables, they may be easier to interpret. The following picture (Figs 13 and 14) shows the results of the jackknife test of variable importance.

**Fig 15:**
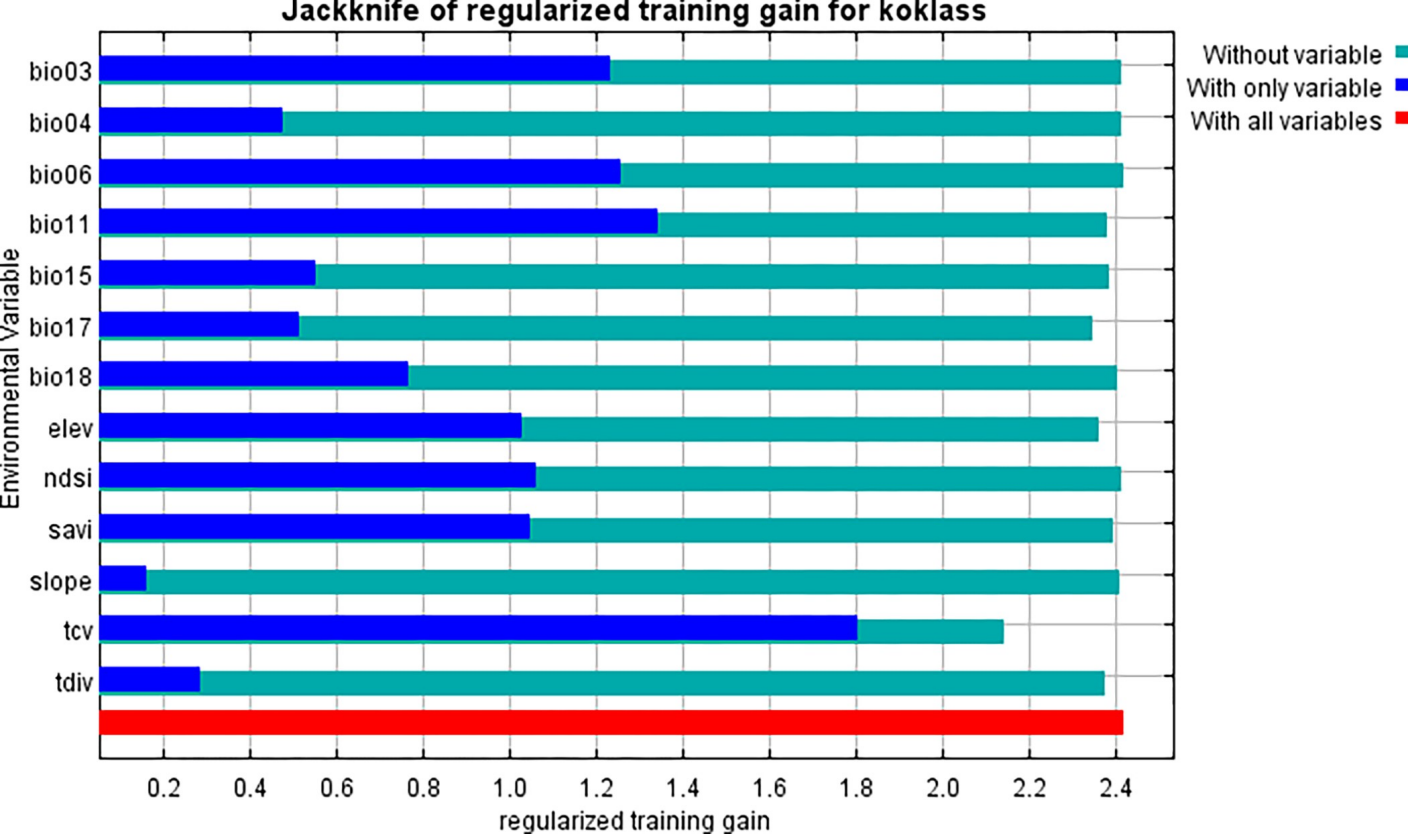
Jacknife analysis of Koklass environmental vs. regularized training gain.

The environmental variable with the highest gain, when used in isolation, is tcv, which therefore appears to have the most useful information by itself. The environmental variable that decreases the gain the most when it is omitted is tcv, which therefore appears to have the most information that isn’t present in the other variables. Values shown are averages over replicate runs. When the predictor variables are associated, variable contributions should be evaluated with caution, just like the variable jackknife. The figures depicted are averages of duplicate runs (Fig 15).

When utilized alone, the environmental variable with the largest gain is tcv (Tree cover), which thus looks to have the most valuable information. When an environmental variable is removed, the gain is reduced the greatest, thus tcv seems to have the most information that isn’t included in the other variables.

**Fig 16:**
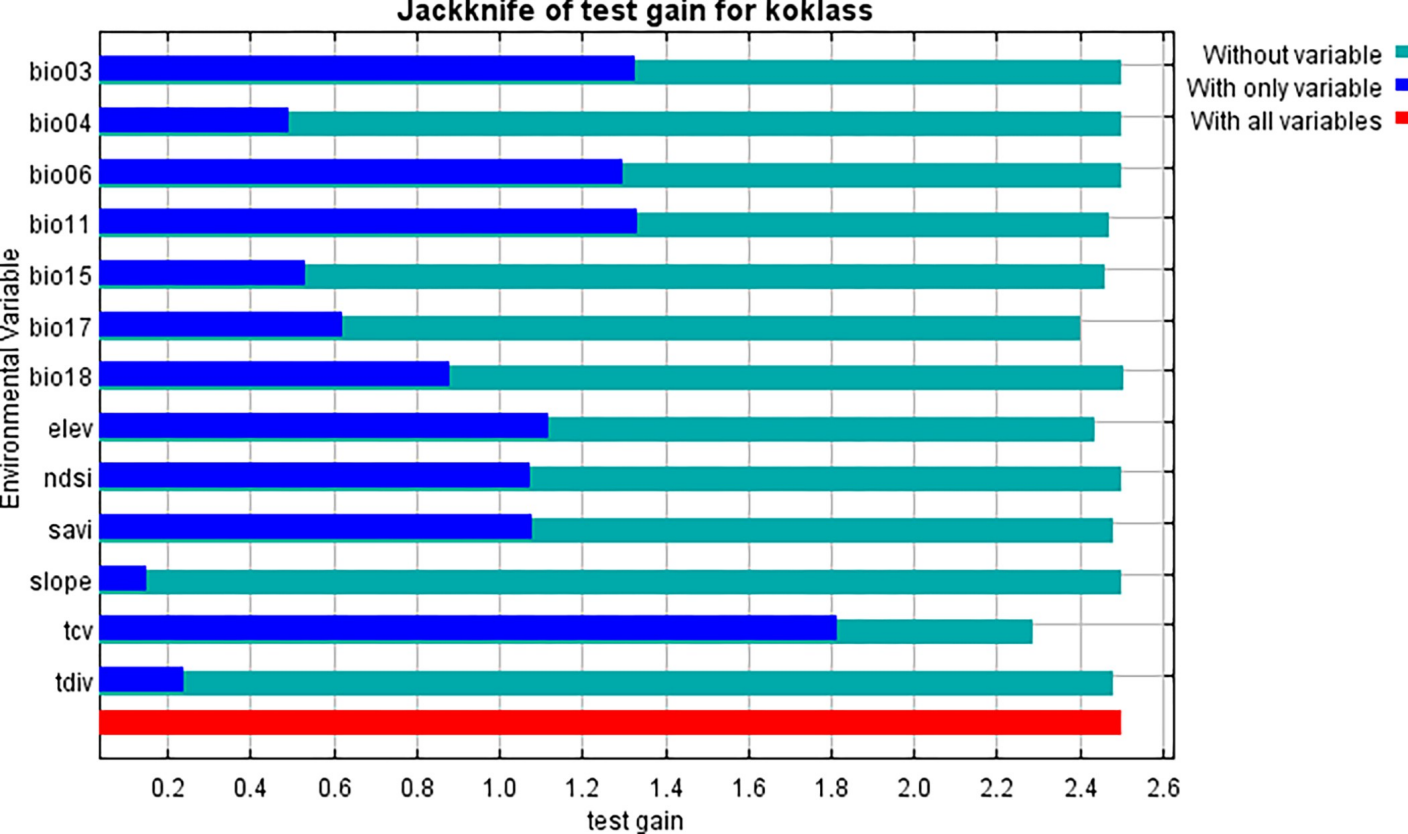
Jacknife of test gain for Koklass.

The image (Fig 16) shows the jackknife test using test gain instead of training gain. It’s important to highlight that when analyzing test data, our evaluations of the most significant factors might change compared to when we examined training data.

**Fig 17:**
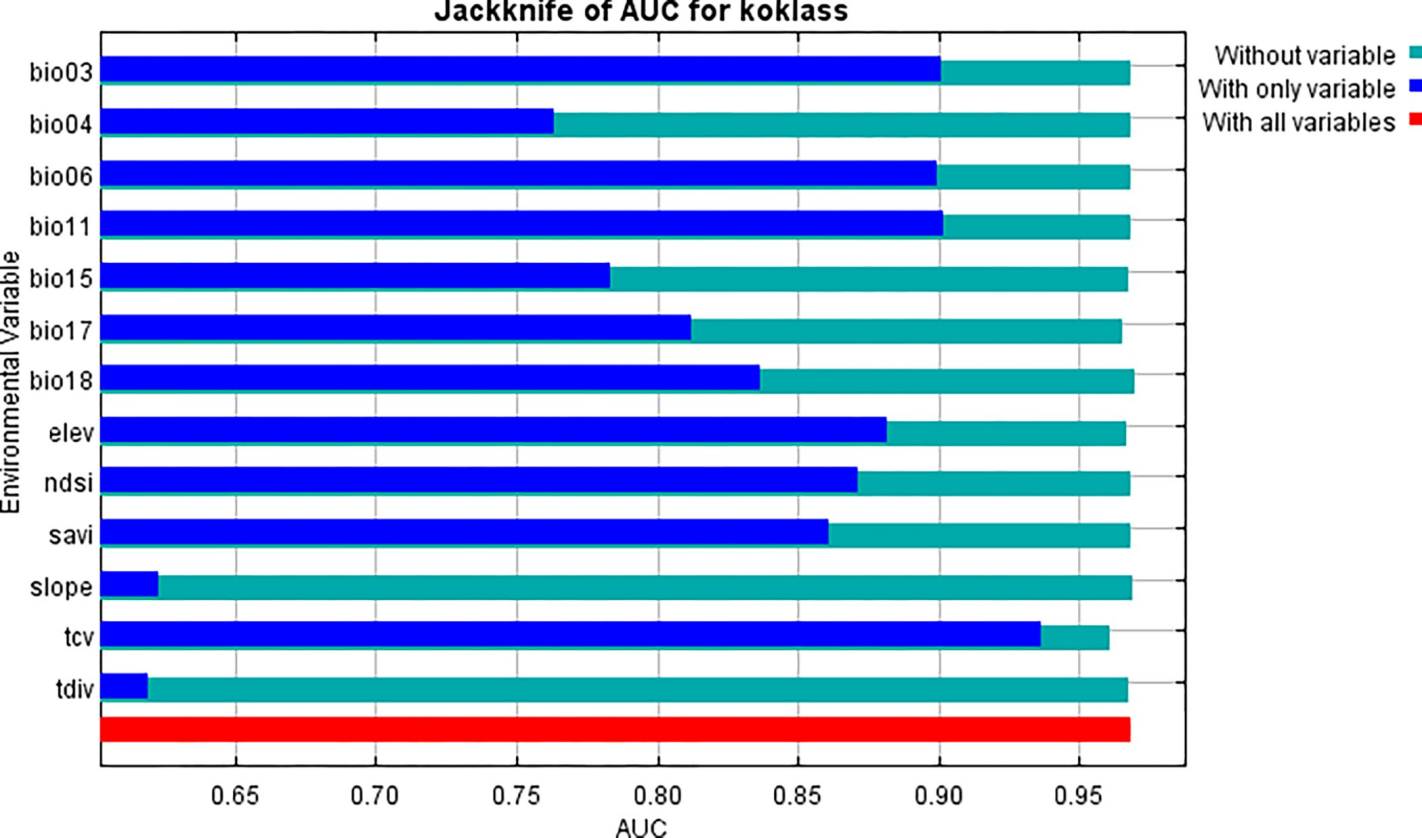
Jacknife analysis of AUC for Koklass.

Finally, using AUC on test data, we do the same jackknife test (Fig 17).

**Fig 18:**
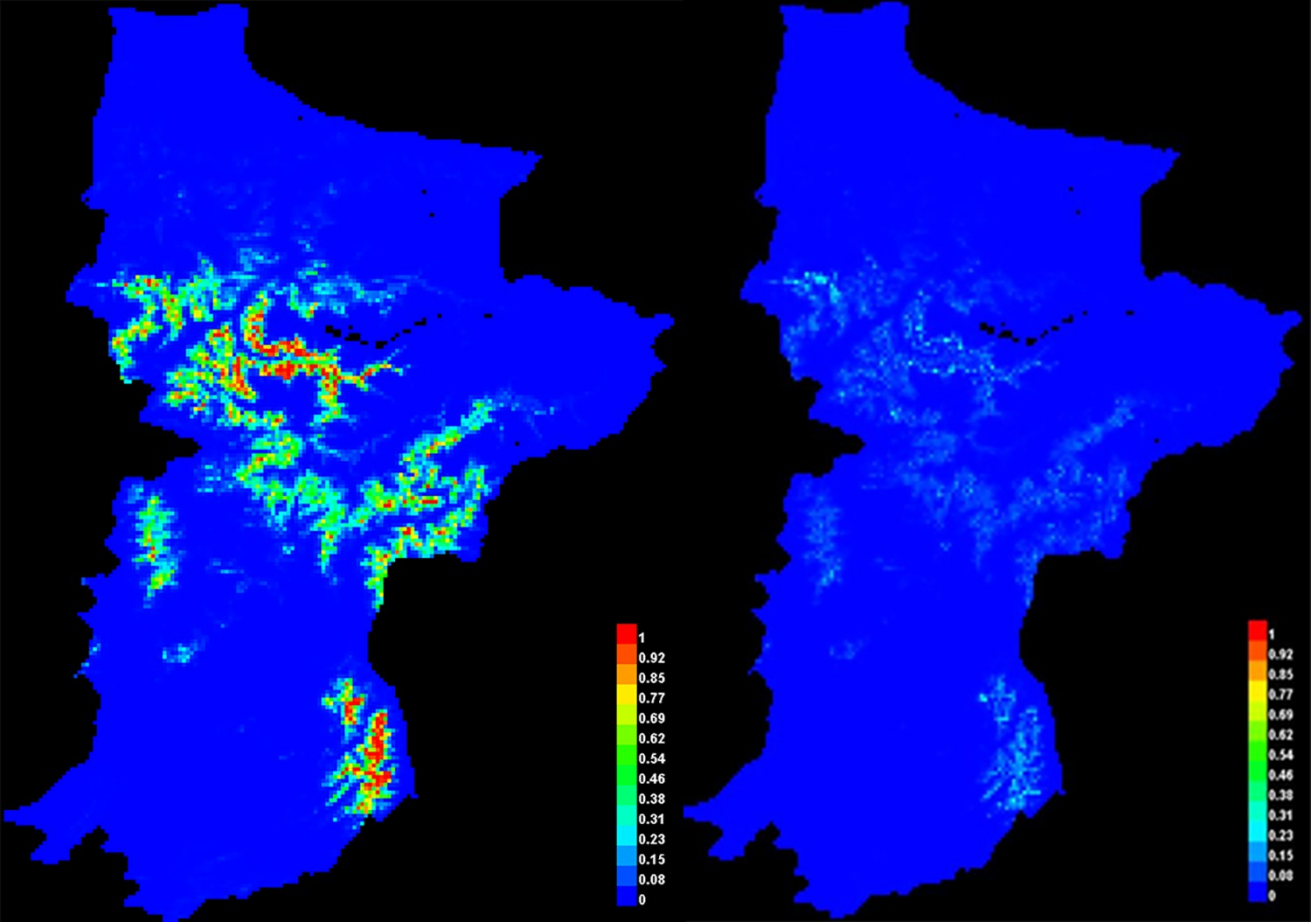
Occurrence points of Koklass in study area.

The following (Fig 18) show the point-wise mean and standard deviation of 3 output grids.

**Fig 19:**
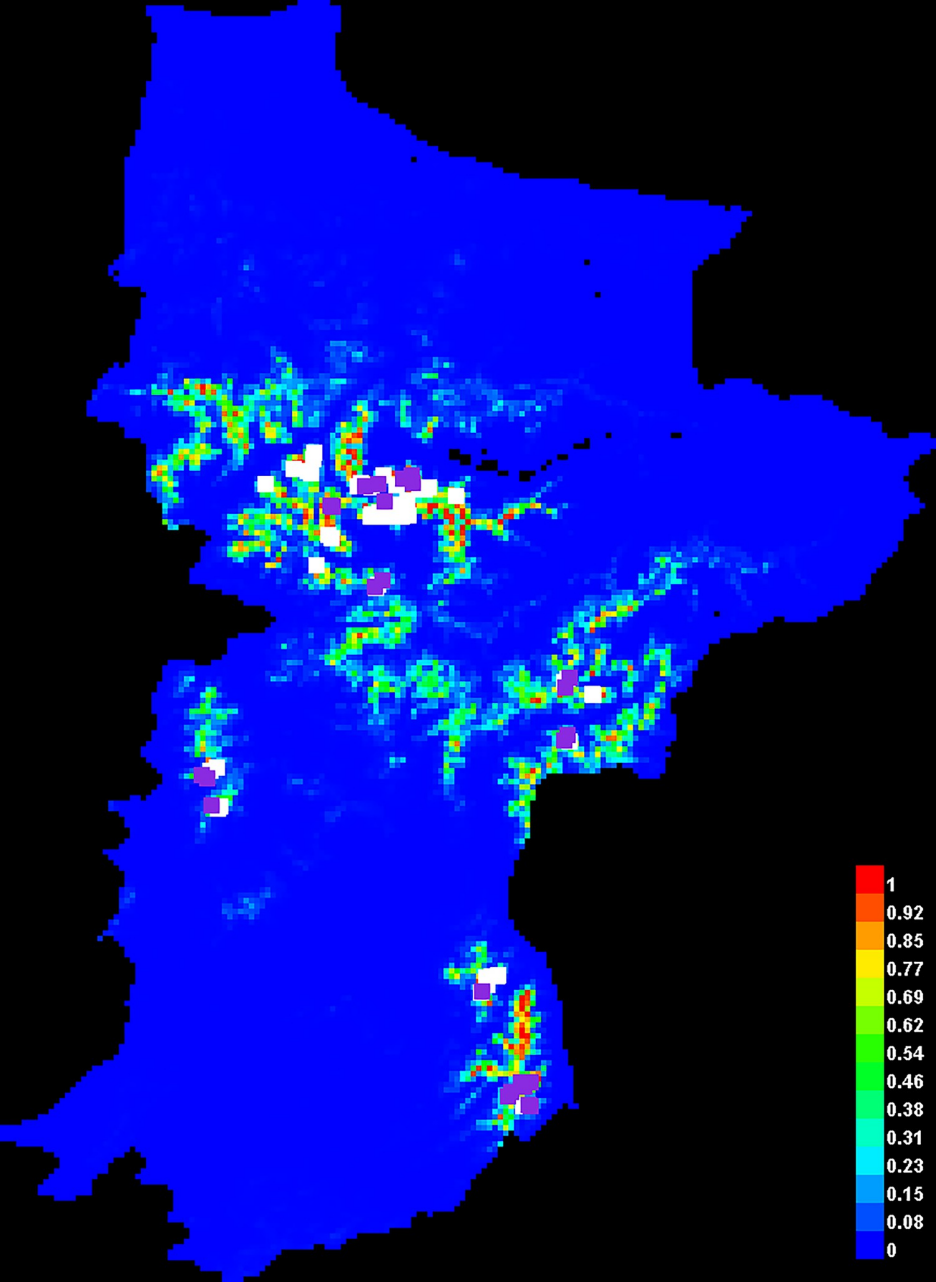
Show suitable area and predicted point of species.

This is a depiction of the Maxent model for Koklass (Fig 19). Areas with warmer hues have better-predicted conditions. The presence sites used for training are shown in white dots, whereas the test locations are shown in violet dots.

**Fig 20:**
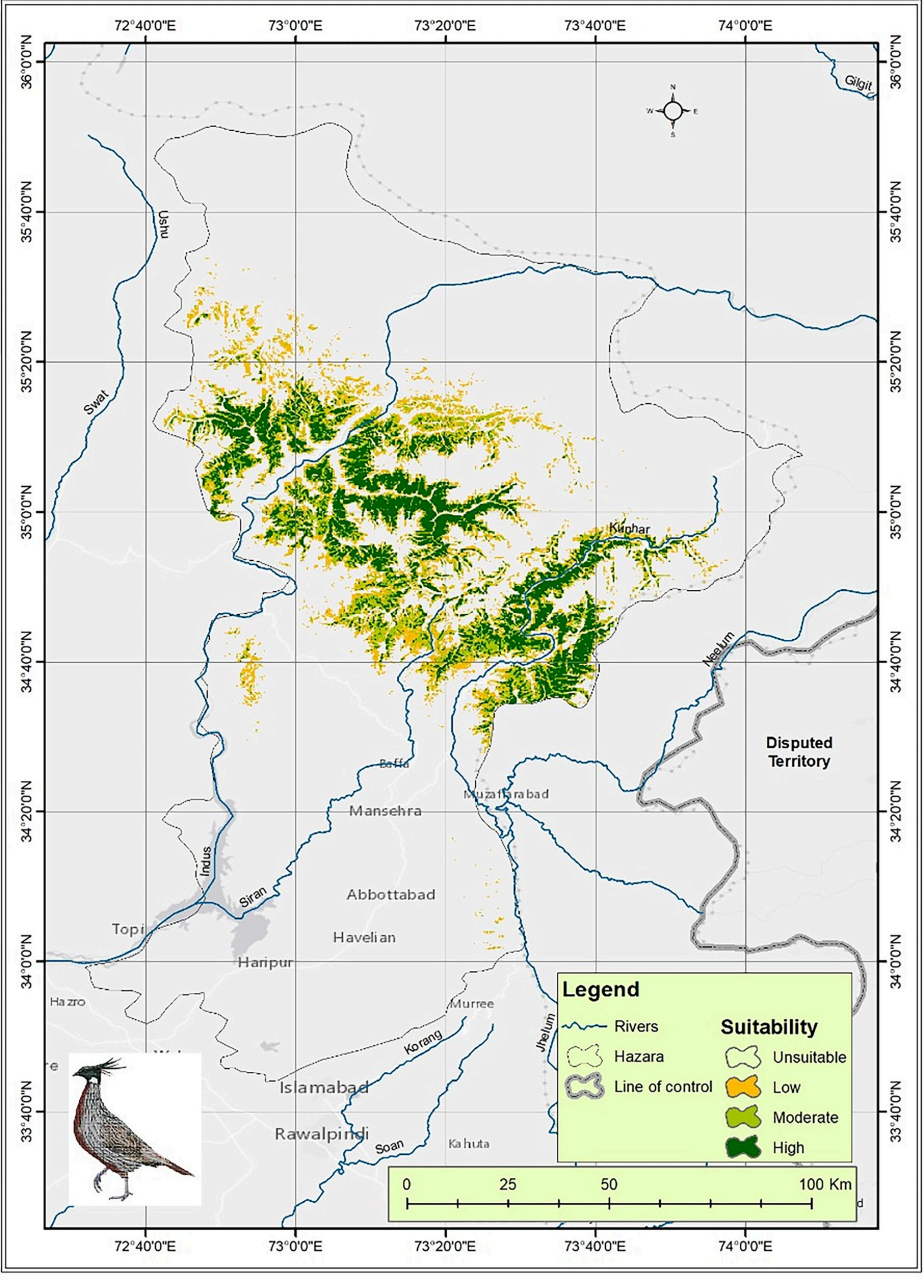
Maxent map of suitable habitat of Koklass pheasant in the study area.

A habitat suitability map (Fig 20) depicting many potential habitat zones (unsuitable, low, moderate, and high suitable) has been created.

The total suitable habitat is 439.6 km^2^_._ The less appropriate habitat zones are primarily due to human activities such as deforestation, domestic cattle grazing, and land expansion. Higher suitability zones have thicker forests, higher canopy cover of trees, and less anthropogenic activity. Although it was also being reported [22].

## Discussion

The study found that the geographical distribution, habitat suitability analysis, and selection of appropriate habitat for the Koklass pheasant are all essential steps in the species’ conservation. Land cover, plant type, and Koklass pheasant sighting locations are all factors to consider. Overall, 69 plant species which include 10 trees, 35 shrubs, 18 herbs, 6 types of grass were present in the habitat.

The investigation underscored the pivotal role of geographical distribution analysis, habitat suitability assessments, and the meticulous selection of habitats crucial for Koklass pheasant conservation initiatives. Paramount factors encompassed land cover intricacies, diverse plant typologies, and precise Koklass pheasant sighting locales. The habitat delineation revealed a diverse assemblage comprising 69 plant species, comprising 10 arboreal species, 35 shrubs, 18 herbaceous varieties, and 6 grass species, collectively shaping the ecological tapestry within the studied habitat. However, it’s noteworthy that a similar methodology was employed by [23], revealing 159 herb species, 21 shrub species, and 15 tree species identified based on behavioral observations. According to the findings, they avoid regions near roads and human settlements. The extinction of this species is attributed to human-caused land encroachment and noise pollution. Results also revealed that the Koklass pheasant is distributed not evenly in the moist temperate forest of Hazara division. The species are found at the elevation 2400 to 3000 m.

Modeling of suitable habitats showed low, moderate, and highly suitable sites for Koklass. The total suitable area of the Koklass pheasant is 439.6 km^2^. The contribution of variable i.e., tcv is most consistently significant across all the other variables as compared to tdiv and bio-03, bio-06, bio-011 contribute comparatively in the model.

The Maxent algorithm’s machine-learning-based mathematics is translated into the statistical language that ecologists can understand. The study investigates several elements of the Maxent environment, with the major focus on a theoretical explanation of maximum entropy. Between two probability densities, the model minimizes the relative entropy (a measure of the distance between distributions), namely (one from the present data and one from the landscape). [24] gave an overview of Maxent’s options and how to use them. The authors begin by providing a statistical description of the basic mechanics of the Maxent method, similar to [25].

## Research implications

In light of the findings from this study, several crucial recommendations emerge for the effective conservation of the Koklass Pheasant and its habitat. Firstly, the Department of Wildlife and Forestry should take proactive measures to enforce legal protection within the areas where these species are distributed. Raising awareness among indigenous populations about the significance of Koklass Pheasant conservation is imperative.

Continued research investigations and regular monitoring of bird species occurrences in the Moist Temperate Forest of Hazara division are essential for informed conservation efforts. Moreover, initiatives aimed at garnering public support, coupled with comprehensive environmental education and awareness programs, should be promoted. Lastly, stringent monitoring and prohibition of land encroachment, clearing for commercial ventures, housing schemes, and other business-oriented purposes are necessary to safeguard the habitat from detrimental human interventions.
